# Smoking, alcohol consumption, and frailty: A Mendelian randomization study

**DOI:** 10.3389/fgene.2023.1092410

**Published:** 2023-02-03

**Authors:** Jiannan Lv, Lianghua Wu, Sheng Sun, Huifang Yu, Zekai Shen, Jun Xu, Jiahao Zhu, Dingwan Chen, Minmin Jiang

**Affiliations:** ^1^ Ganyao Town Hospital of Jiashan County, Jiaxing, China; ^2^ Majin Town Hospital of Kaihua County, Quzhou, China; ^3^ School of Public Health, Hangzhou Medical College, Hangzhou, China; ^4^ Key Laboratory of Pollution Exposure and Health Intervention of Zhejiang Province, Shulan International Medical College, Zhejiang Shuren University, Hangzhou, China

**Keywords:** smoking, alcohol, frailty, Mendelian randomization, causality

## Abstract

**Background:** Tobacco smoking and alcohol consumption have been associated with frailty in observational studies. We sought to examine whether these associations reflect causality using the two-sample Mendelian randomization (MR) design.

**Methods:** We used summary genome-wide association statistics for smoking initiation (*N* = 2,669,029), alcohol consumption (*N* = 2,428,851), and the frailty index (FI, *N* = 175,226) in participants of European ancestry. Both univariable and multivariable MR were performed to comprehensively evaluate the independent effects of smoking and alcohol consumption on the FI, accompanied by multiple sensitivity analyses. Results were verified using lifetime smoking and alcohol use disorder. Reverse direction MR was undertaken to assess the potential for reverse causation.

**Results:** Genetic predisposition to smoking initiation was significantly associated with increased FI (univariable MR: *β* = 0.345; 95% confidence interval [CI] = 0.316 to 0.374; *p* = 1.36E-113; multivariable MR: *β* = 0.219; 95% CI = 0.197 to 0.241; *p* = 2.44E-83). Genetically predicted alcohol consumption showed a suggestive association with the FI (univariable MR: *β* = −0.090; 95% CI = −0.151 to −0.029; *p* = 0.003; multivariable MR *β* = −0.153; 95% CI = −0.212 to −0.094; *p* = 2.03E-07), with inconsistent results in sensitivity analyses. In complementary analysis, genetic predicted lifetime smoking, but not alcohol use disorder was associated with the FI. There is no convincing evidence for reverse causation.

**Conclusion:** The present MR study supported smoking as a causal risk factor of frailty. Further research is warranted to investigate whether alcohol consumption has a causal role in frailty.

## Introduction

Frailty is a complex geriatric syndrome characterized by reduced physiological reserve and increased vulnerability to stressor events due to dysfunctions across multiple physiological systems during a lifetime (Clegg et al., 2013). With population ageing, frailty is increasingly prevalent worldwide and exerts a major public health burden for its close associations with many adverse health outcomes such as multimorbidity, disability, and excess mortality (Clegg et al., 2013).

Cigarette smoking and alcohol use have received particular attention as promising targets in the prevention and management of frailty, because both are potentially modifiable lifestyle factors (Mello Ade et al., 2014). Previous observational studies have identified a positive relationship of smoking with incident frailty among older adults (Kojima et al., 2015), but paradoxical findings regarding the role of alcohol consumption were reported. Some studies showed that heavy alcohol use was related to lower risk of developing frailty compared with never alcohol use (Kojima et al., 2018a), while some others found no such benefits of alcohol consumption (Seematter-Bagnoud et al., 2014) or an apparently detrimental effect (Strandberg et al., 2018). Moreover, current evidence linking smoking and alcohol consumption to frailty mainly originates from conventional observational studies, which are vulnerable to confounding (e.g., socioeconomic status influencing smoking, alcohol use, and frailty) and reverse causation (e.g., drinkers reducing alcohol consumption when frail, known as the “sick quitter” effect). As such, the causal nature of the associations of smoking and alcohol consumption with frailty remains elusive.

When randomized controlled trials are not feasible or ethical to conduct, Mendelian randomization (MR) provides an alternative means to assess the causal effect of the exposure on the outcome by utilizing genetic variants as instruments (Smith and Ebrahim, 2003). MR can diminish the issues of unmeasured confounding and reverse causation, because genetic variants are randomly assorted at conception and are not modified by the progression of disease (Smith and Ebrahim, 2003). Recently, the MR approach has been successfully applied to shed light on the causal roles of smoking and alcohol consumption in a wide range of health outcomes (Rao et al., 2021; Larsson and Burgess, 2022; van de Luitgaarden et al., 2022; Zhang and Baranova, 2022; Baranova et al., 2023). Herein, we applied the MR design to explore the potential causal associations of smoking as well as alcohol consumption with frailty.

## Methods

### Study design

**FIGURE 1:**
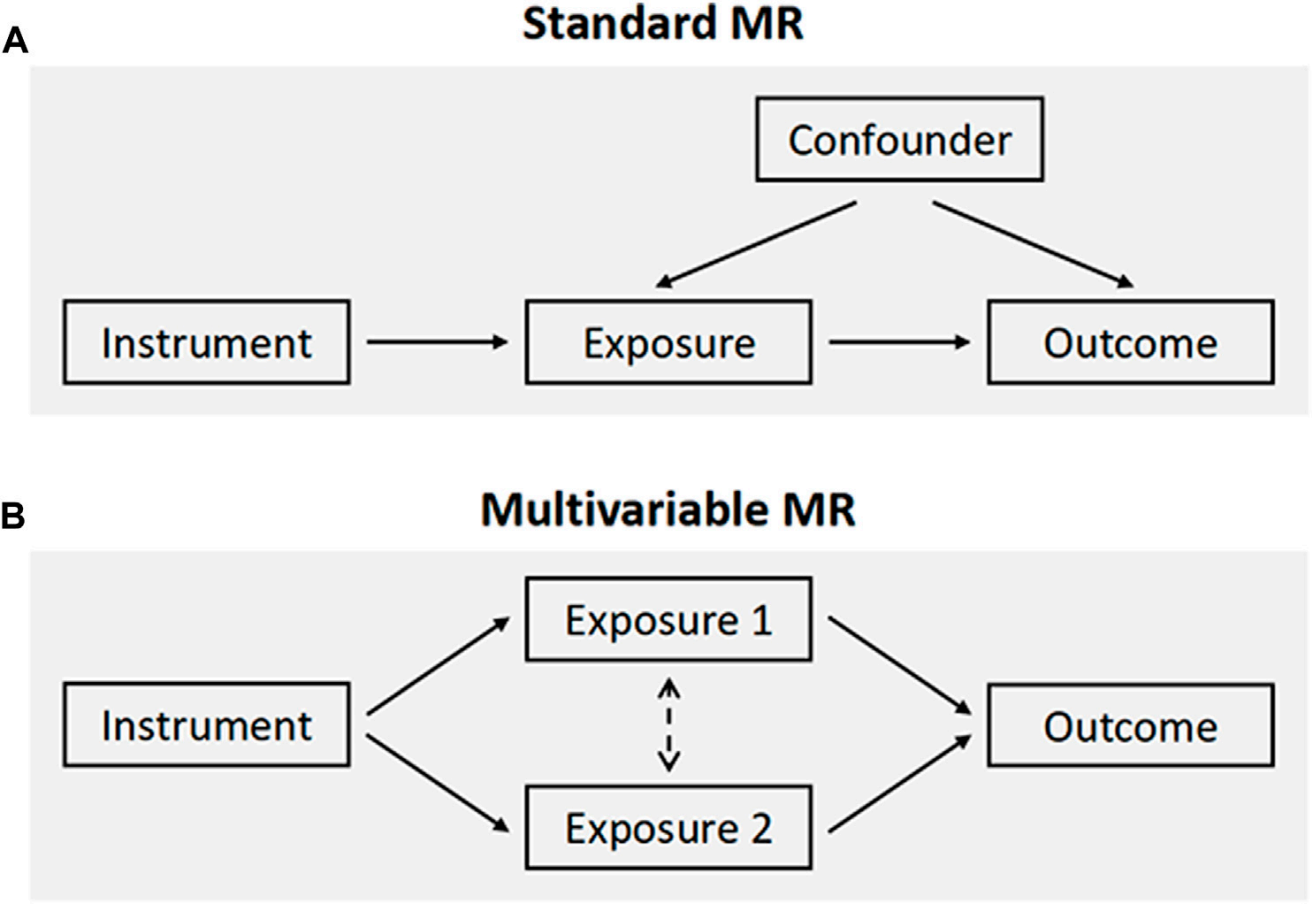
Schematic overview of the study design. **(A)** The standard MR framework. MR relies on three core assumptions for causal inference: the genetic variants are associated with the exposure (relevance assumption), are independent of confounders (independence assumption), and affect the outcome through the exposure of interest only (exclusion restriction assumption, also known as the “no pleiotropy”). **(B)** Multivariable MR, an extension of univariable MR, leverages genetic instruments associated with multiple correlated exposures to jointly estimate the independent effect of each of the exposures on the outcome. MR, Mendelian randomization.

An overview of the MR framework was illustrated in Figure 1A. To comprehensively appraise the causal role of both smoking and alcohol consumption in frailty, we first performed univariable MR analyses. Considering a moderate genetic correlation between smoking and alcohol use (r_g_ = 0.34) (Liu et al., 2019), multivariable MR was applied to simultaneously assess the independent effects of each (Figure 1B). Additionally, we carried out reverse direction MR to explore the potential for reverse causation. All MR analyses were conducted in a two-sample approach using publicly available summary statistics, and thus no additional ethical approval or informed consent would be required. This study was reported in alignment with the STROBE-MR guideline (Skrivankova et al., 2021).

### Data source for frailty

Summary statistics for frailty, measured by the frailty index (FI) phenotype, were obtained from a recent meta-analysis of genome-wide association studies (GWAS) in the United Kingdom Biobank and Swedish TwinGene, which includes 175,226 participants of European ancestry (Atkins et al., 2021). The FI is a continuous measure and presented as a proportion of the sum of all age-related health deficits with over 40 components, covering a range of physiological and mental health domains (Mitnitski et al., 2001; Atkins et al., 2021). As a proxy of overall health, the FI has been validated to strongly predict many adverse health outcomes and shown to be more suitable for assessing frailty at younger age than other measures (Blodgett et al., 2015; Theou et al., 2016; Williams et al., 2019).

### Selection of genetic instruments

We selected genetic instruments for smoking initiation and alcohol consumption from the largest GWAS meta-analysis to date performed by the GWAS and Sequencing Consortium of Alcohol and Nicotine use (GSCAN), involving 2,669,029 individuals of European ancestry (Saunders et al., 2022). Smoking initiation is a binary phenotype indicating whether an individual ever being a regular smoker during the life course. Alcohol consumption was defined as the average amount of drinks an individual reported drinking per week, regardless of types of alcohol. A total of 1,752 conditionally independent single-nucleotide polymorphisms (SNPs) associated with smoking initiation and 501 conditionally independent SNPs with alcohol consumption at genome-wide significance (*p* < 5 × 10^−9^) were identified based on sequential forward selection (for more details see the original GWAS) (Saunders et al., 2022). These genome-wide significant SNPs explained 10% and 1% of the phenotypic variance in smoking initiation and alcohol consumption, respectively, and were treated as robust instruments with *F*-statistics >10 in our MR study. In multivariable MR analysis, we did linkage disequilibrium clumping for the combined set of SNPs for both smoking initiation and alcohol consumption using PLINK (r^2^ = 0.1 and distance = 10,000 kb) with a European ancestry reference panel from the 1,000 Genome Project, resulting in 1,878 independent SNPs used as genetic instruments.

### Statistical analysis

All SNPs were harmonized for the exposure and the outcome by alleles to ensure the alignment of effect. Where possible, instrumental SNPs for the exposure absent in the outcome data sets were proxied using variants in linkage disequilibrium (r^2^ > 0.8).

Given the significant heterogeneity across SNP effects, we applied multiplicative random-effects model in inverse-variance weighted (IVW) approach as the principal analysis in both univariable and multivariable MR (Burgess et al., 2013; Sanderson et al., 2019). The covariance between SNP effects on each exposure was fixed at zero in the multivariable setting. The IVW approach provides a weighted average of SNP effects where the intercept is constrained to zero (Burgess et al., 2013). However, results can be biased if instrumental SNPs show horizontal pleiotropy, a major source of bias in the MR setting. We therefore compared univariable IVW estimates with a suite of other well-established MR methods relatively robust to pleiotropy, including the weighted median (Bowden et al., 2016), weighted mode (Hartwig et al., 2017), and MR-Egger regression (Bowden et al., 2015). Multivariable median-based method and multivariable MR-Egger were implemented for this purpose in multivariable MR analyses. These methods rely on different assumptions such that a consistent effect across multiple methods enables to draw conclusions about causality with greater persuasiveness.

To further examine the robustness of results, we checked for evidence of heterogeneity (a potential indicator of pleiotropy) in IVW estimators using the Cochran’s Q statistic. The MR Egger intercept test was used to indicate the presence of directional pleiotropy (Bowden et al., 2015). The Radial MR was used to identify outliers and all MR analyses were repeated after removing these outlying SNPs (Bowden et al., 2018). Leave-one-out analyses were undertaken to evaluate whether the overall estimates were driven by a single SNP.

### Complementary analysis

As secondary phenotypes, we evaluated lifetime smoking (a continuous measure that takes into account smoking initiation, duration, heaviness, and cessation) and alcohol use disorder for validation. Genetic instruments for lifetime smoking were constructed using 126 conditionally independent SNPs from a GWAS in the United Kingdom Biobank (*N* = 462,690) (Wootton et al., 2020). For alcohol use disorder, we selected 24 conditionally independent SNPs as genetic instruments from a GWAS meta-analysis of Million Veterans Program and Psychiatric Genomics Consortium (Ncase = 57,564, Ncontrol = 256,395) (Zhou et al., 2020).

To test for reverse causation (i.e., whether frailty influences smoking and alcohol intake), we did MR analyses in the opposite direction using 14 lead SNPs at distinct loci associated with the FI as instruments (Atkins et al., 2021; Zhu et al., 2022). In a further complementary analysis, we repeated MR analyses in both directions using the Steiger filtering (Hemani et al., 2017). This method removes instrumental SNPs that explain more variance of the outcome than the exposure to ensure the orientation of inferred causal relationships.

All statistical analyses were performed using the “TwoSampleMR,” “MendelianRandomizaiton,” and “MRPRESSO” packages in R version 3.6.3. Significant associations were defined as the MR estimates passing the nominal significance (*p* < 0.05), showing the same direction of effect across sensitivity analyses, and presenting limited influence of horizontal pleiotropy based on the MR-Egger intercept.

## Results

The results of IVW method showed that genetic predisposition to smoking initiation was positively associated with the FI in both univariable MR (*β* = 0.345; 95% confidence interval [CI] = 0.316 to 0.374; *p* = 1.36E-113) and multivariable MR conditioning for alcohol consumption (*β* = 0.219; 95% CI = 0.197 to 0.241; *p* = 2.44E-83) (Table 1). Sensitivity analyses using multiple pleiotropy-robust methods yield similar results for smoking initiation. For the IVW method, genetically predicted alcohol consumption showed a negative association with the FI in both univariable MR (*β* = −0.090; 95% CI = −0.151 to −0.029; *p* = 0.003) and multivariable MR (*β* = −0.153; 95% CI = −0.212 to −0.094; *p* = 2.03E-07) (Table 2). However, univariable pleiotropy-robust methods presented null associations and inconsistent direction of effect. The negative association between alcohol consumption and the FI was stable in multivariable sensitivity analyses. There was evidence of directional pleiotropy for alcohol consumption (*p* = 0.001), but not for smoking initiation (*p* = 0.651) using the MR-Egger intercept. Cochran’s Q statistic detected notable heterogeneity across the SNP effects (all *p* < 0.001). After removing potential outliers in Radial MR, results were largely unchanged (Tables 1, 2), and heterogeneity or pleiotropy was no longer detected (all *p* > 0.05). Leave-one-out analyses demonstrated that no single SNP drove these associations. Scatter plots are presented in Supplementary Figures S1, S2.

**TABLE 1:** MR results for the effect of smoking initiation on the frailty index.

Method	Beta	95% CI	*p*-Value
Univariable MR			
IVW (multiplicative random effects)	0.345	0.316, 0.374	1.36E-113
Weighted median	0.312	0.275, 0.349	1.78E-62
Weighted mode	0.212	0.085, 0.339	1.08E-03
MR-Egger	0.325	0.235, 0.415	1.51E-12
Radial MR (IVW)	0.339	0.315, 0.363	1.70E-178
Multivariable MR adjusting for alcohol consumption			
Multivariable IVW	0.219	0.197, 0.241	2.44E-83
Multivariable median-based	0.237	0.212, 0.262	3.26E-77
Multivariable MR-Egger	0.217	0.172, 0.262	1.40E-21

CI, confidence interval; IVW, inverse-variance weighted; MR, Mendelian randomization; MR-PRESSO, MR-Pleiotropy RESidual Sum and Outlier; SE standard error.

**TABLE 2:** MR results for the effect of alcohol consumption on the frailty index.

Method	Beta	95% CI	*p*-Value
Univariable MR			
IVW (multiplicative random effects)	−0.090	−0.151, −0.029	0.003
Weighted median	−0.028	−0.099, 0.043	0.427
Weighted mode	0.009	−0.077, 0.095	0.843
MR-Egger	0.099	−0.028, 0.226	0.127
Radial MR (IVW)	−0.118	−0.163, −0.073	2.26E-07
Multivariable MR adjusting for smoking initiation			
Multivariable IVW	−0.153	−0.212, −0.094	2.03E-07
Multivariable median-based	−0.210	−0.279, −0.141	2.37E-09
Multivariable MR-Egger	−0.154	−0.213, −0.095	2.22E-07

CI, confidence interval; IVW, inverse-variance weighted; MR, Mendelian randomization; MR-PRESSO, MR-Pleiotropy RESidual Sum and Outlier.

In a complementary analysis using secondary phenotypes, we observed a strong association of genetic predicted lifetime smoking (*β* = 0.382; 95% CI = 0.316 to 0.448, *p* = 1.40E-29), but not alcohol use disorder with the FI (Supplementary Table S1). In the reverse direction MR analysis, there was some evidence that higher genetically predicted FI may lead to smoking initiation (*β* = 0.385; 95% CI = 0.056 to 0.714, *p* = 0.005), but uncertainty existed due to inconsistent results across different methods (Supplementary Table S2). Results were largely unchanged in a further analysis using Steiger filtering.

## Discussion

To our knowledge, this is the first study to determine the causal associations of smoking as well as alcohol consumption with frailty based on the MR framework. We found robust evidence supporting a causal effect of cigarette smoking on increasing frailty. In addition, there was suggestive evidence to support the protective role of alcohol consumption in frailty.

Most of the previous longitudinal studies suggested a detrimental effect of smoking on frailty (Woods et al., 2005; Ottenbacher et al., 2009; Wang et al., 2013; Etman et al., 2015; Kojima et al., 2018b). Ottenbacher et al., found smoking status as a predictor of frailty during the 10-year follow-up in 2,049 Mexican Americans (Ottenbacher et al., 2009). Woods et al. conducted a prospective study in 40,657 women and reported that smoking was strongly associated with the risk of developing frailty (Woods et al., 2005). In the analysis of 3,257 community-dwelling people with a 15-year follow-up, Wang et al. found that male smokers were frailer than male non-smokers, but no such difference was seen in women (Wang et al., 2013). A systematic review of five studies supported smoking as a risk factor of worsening frailty status in older adults but highlighted that a causal relationship cannot be established, as only a limited number of confounding covariates have been adjusted (Kojima et al., 2015). Only one study of 3,018 community-dwelling older adults in China failed to show a significant association of smoking with higher risk of frailty after over 2 years of follow-up (Lee et al., 2014), which may be the results of low statistical power. Our study strengthened the evidence for the causal effect of smoking on increasing frailty by using the MR approach, which is less susceptible to confounding bias than traditional observational designs. However, due to the use of summary-level data, we were unable to explore the sex-specific associations, which warrant further investigation.

The mechanisms by which smoking worsens subsequent frailty status are unknown but are in line with the understanding that tobacco smoke contains various kinds of toxic chemicals and compounds affecting nearly every organ and tissue (NHS, 2021). Smoking has been associated with a spectrum of physical and mental disorders (NHS, 2021), all of which may further contribute to increased risk of developing frailty. Furthermore, chronic systemic inflammation and oxidative stress may be common pathways mediating the relationship between tobacco smoking and frailty (Yanbaeva et al., 2007). In terms of public health implications, our findings may be in favor of smoking cessation programs as an effective strategy to reduce or delay the onset of frailty and its resultant poor outcomes. This potential benefit of smoking cessation was supported by a recent prospective study reporting no significant difference in incident frailty between past smokers and never smokers (Kojima et al., 2018b).

Longitudinal studies looking into alcohol use and frailty risk are more mixed in their results than in the case of smoking and frailty. In a meta-analysis of four prospective studies, heavy drinkers were reported to have lower incident frailty compared with non-drinkers in middle-aged and older adults, but follow-up time ranged from 2 to 3.3 years among included studies (Kojima et al., 2018a). On the contrary, a more recent study, the Helsinki Businessmen Study, using the 30-year follow-up data implicated that high alcohol consumption, but not zero, in midlife predicted both frailty and pre-frailty in old age (Strandberg et al., 2018). Data from the Lausanne cohort showed no significant incident vulnerability in heavy drinkers compared with the light-to-moderate drinkers (Seematter-Bagnoud et al., 2014). The conflicting findings may be attributed to uncontrolled confounding, reverse causation, or different definitions of alcohol consumption and frailty. Overcoming several weaknesses inherent in observational studies, the present MR study provided suggestive evidence for a causal protective role of alcohol consumption on the FI. However, this finding should be interpreted with caution, due to the inconsistent results across sensitivity analyses and a null association with alcohol use disorder. The potential benefits of alcohol against frailty may be explained by social components, which were included in some multidimensional frailty criteria (Gobbens et al., 2010). Alcohol is often consumed socially, and moderate consumption was shown to facilitate social bonding (Sayette et al., 2012) and may help reinforce social support or network and prevent social isolation.

The major strength of this study included the ability of two-sample MR design to improve the causal inference in a cost-efficient manner and the application of multivariable MR to separate the effects of smoking and alcohol consumption. Inevitably, there are several limitations. The most notable challenge in MR is horizontal pleiotropy whereby genetic variants affect the outcome through a pathway other than the exposure of interest. While we leveraged a large number of genetic variants associated with smoking and alcohol consumption as strong instruments, these variants are not well characterized. It is possible that pleiotropic variants that impact pathways outside of smoking or alcohol were introduced. Non-etheless, our sensitivity analyses incorporating multiple pleiotropy-robust MR methods provided largely similar results. Another limitation is that the current summary-level MR precluded us to assess the potentially non-linear relationships. Several studies have revealed a possible J- or U-shaped association between alcohol consumption and frailty, with lower frailty risk in light-to-moderate drinkers compared with non- and heavy drinkers (Woods et al., 2005; Etman et al., 2015). Therefore, future individual-level MR investigations using non-linear methods are warranted. Moreover, our analyses comprised participants of predominantly European ancestry, thereby restricting the generalization of findings to other ethnic populations. However, population stratification bias was expected to be largely eliminated.

In conclusion, the present MR study supported smoking as a causal risk factor of frailty. Further research is warranted to investigate whether alcohol consumption has a causal role in frailty. Further understanding of these associations could be beneficial for facilitating the development of prevention strategies.

## Data Availability

The original contributions presented in the study are included in the article/Supplementary Material, further inquiries can be directed to the corresponding authors.
