# Supplementary material for: Smoking, alcohol consumption, and frailty: A Mendelian randomization study
Source: Front Genet. 2023 Feb 3;14:1092410. doi: 10.3389/fgene.2023.1092410 (PMC9935614; doi:10.3389/fgene.2023.1092410)
Supplement: Supplementary file 1 [file Table1.DOCX]

Supplementary Materials for

**Smoking, alcohol consumption, and frailty: a Mendelian randomization study**

**Contents**

**Supplementary Table 1** MR results for the effect of lifetime smoking and alcohol use disorder on the frailty index……………………..……………………….…….…...…2

**Supplementary Table 2** MR results for the effect of the frailty index on smoking initiation and alcohol consumption……………………………………………....….…3

**Supplementary Figure 1** Scatter plot for the effect of smoking initiation on the frailty index………………………………………………………….……….......………...…4

**Supplementary Figure 2** Scatter plot for the effect of alcohol consumption on the frailty index………………………………………..………………....…..........….……5

| **Supplementary Table 1** MR results for the effect of lifetime smoking and alcohol use disorder on the frailty index. | | | | |
| --- | --- | --- | --- | --- |
| Exposure | Method | Beta | SE | P |
| Lifetime smoking | IVW | 0.382 | 0.034 | 1.40E-29 |
|  | Weighted median | 0.314 | 0.038 | 1.01E-16 |
|  | Weighted mode | 0.254 | 0.086 | 3.82E-03 |
|  | MR-Egger | 0.219 | 0.133 | 0.103 |
|  | Radial MR (IVW) | 0.386 | 0.028 | 1.52E-43 |
| Alcohol use disorder | IVW | 0.092 | 0.056 | 0.101 |
|  | Weighted median | 0.054 | 0.042 | 0.198 |
|  | Weighted mode | 0.047 | 0.045 | 0.302 |
|  | MR Egger | -0.007 | 0.101 | 0.943 |
|  | Radial MR (IVW) | 0.056 | 0.034 | 0.100 |
| Abbreviations: IVW, inverse-variance weighted; MR, Mendelian randomization; SE, standard error. | | | | |

| **Supplementary Table 2** MR results for the effect of the frailty index on smoking initiation and alcohol consumption. | | | | |
| --- | --- | --- | --- | --- |
| Outcome | Method | Beta | SE | P |
| Smoking initiation | IVW | 0.385 | 0.168 | 0.022 |
|  | Weighted median | 0.191 | 0.078 | 0.014 |
|  | Weighted mode | 0.188 | 0.089 | 0.056 |
|  | MR-Egger | -0.097 | 0.787 | 0.904 |
|  | Radial MR (IVW) | 0.410 | 0.111 | 2.89E-06 |
| Alcohol consumption | IVW | 0.020 | 0.057 | 0.724 |
|  | Weighted median | 0.054 | 0.040 | 0.181 |
|  | Weighted mode | 0.056 | 0.049 | 0.270 |
|  | MR Egger | 0.210 | 0.265 | 0.443 |
|  | Radial MR (IVW) | 0.041 | 0.031 | 0.186 |
| Abbreviations: IVW, inverse-variance weighted; MR, Mendelian randomization; SE, standard error. | | | | |

**Supplementary Figure 1** Scatter plot for the effect of smoking initiation on the frailty index.


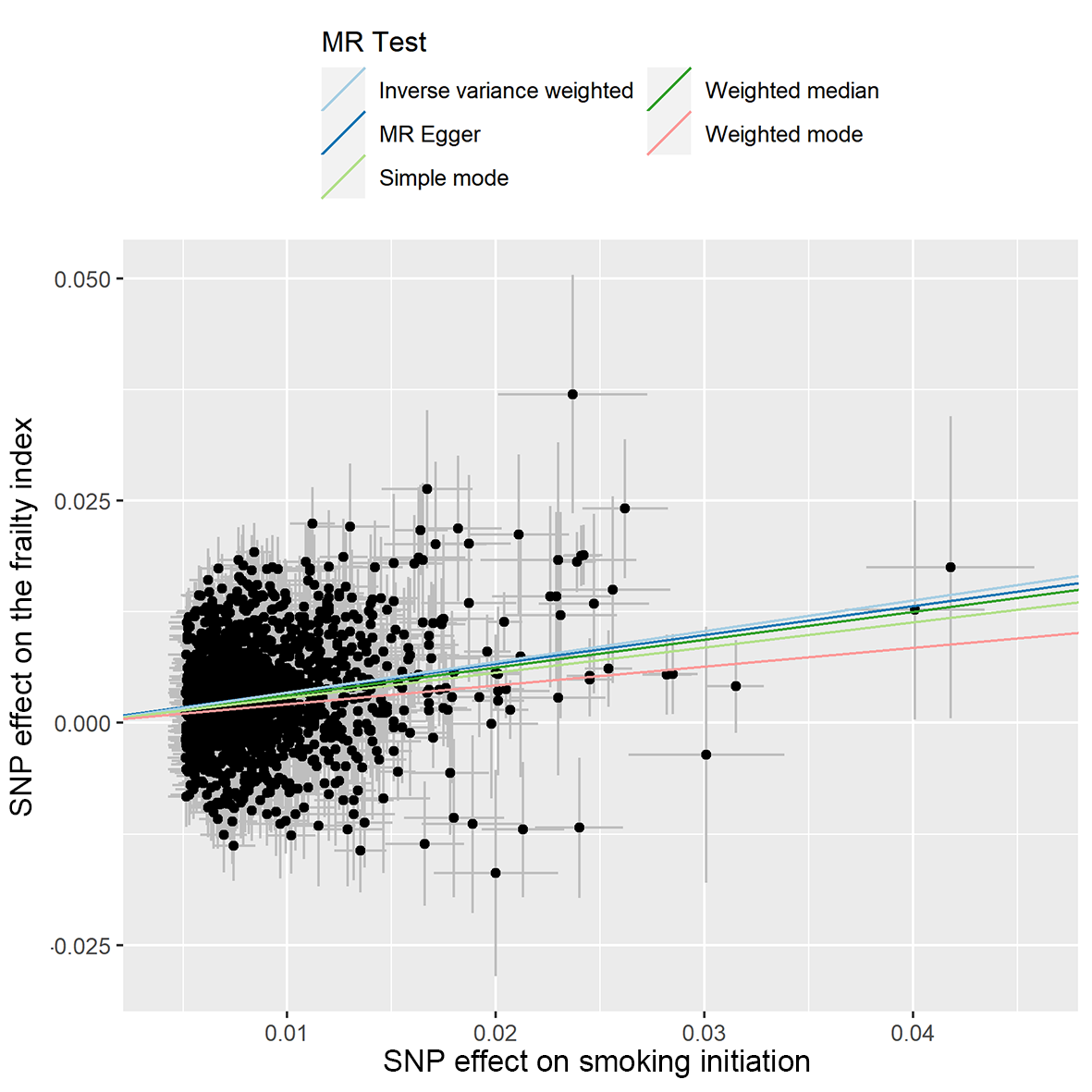


**Supplementary Figure 2** Scatter plot for the effect of alcohol consumption on the frailty index.

**
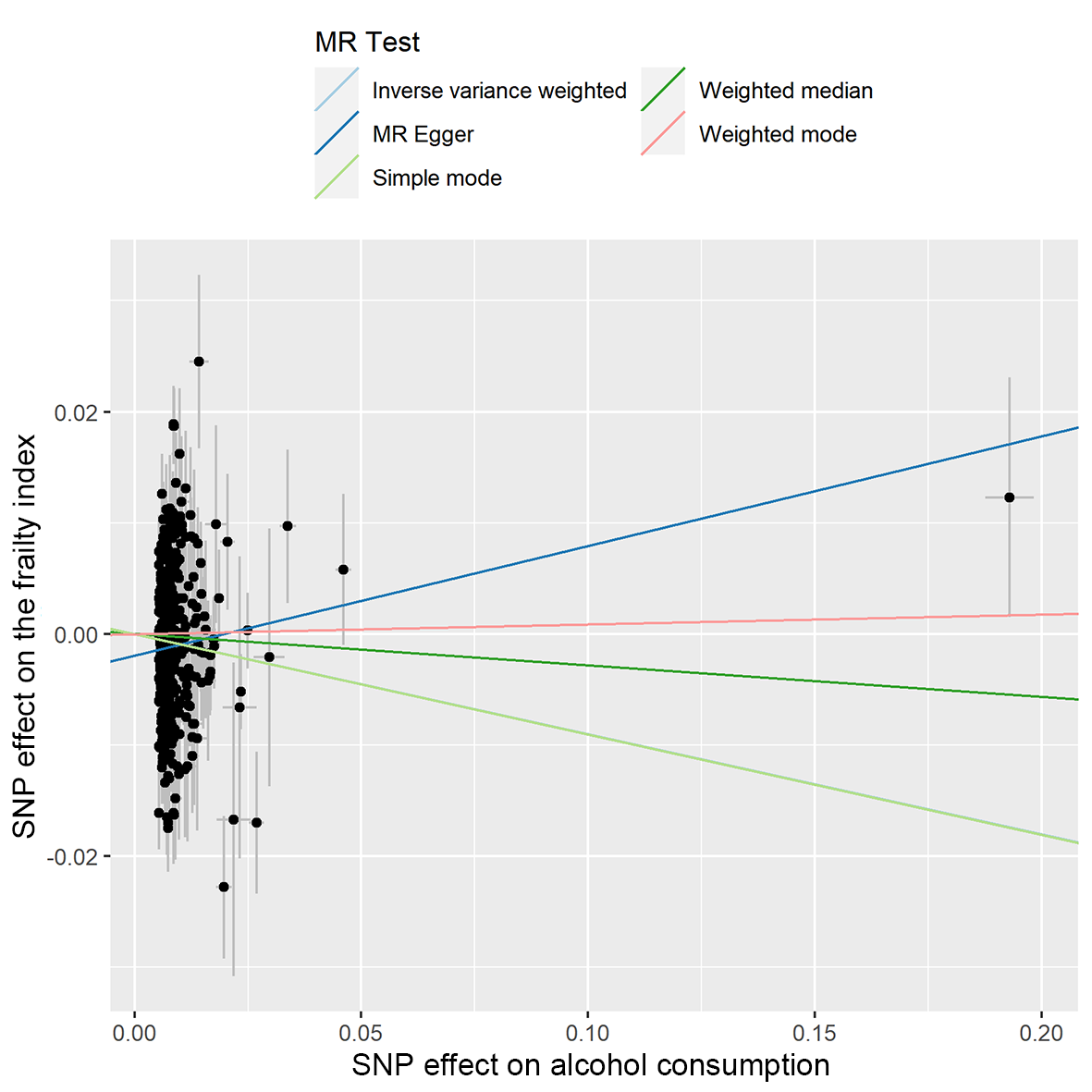
**
